# Generation of Osteosarcomas from a Combination of Rb Silencing and c‐Myc Overexpression in Human Mesenchymal Stem Cells

**DOI:** 10.5966/sctm.2015-0226

**Published:** 2016-09-07

**Authors:** Jir‐You Wang, Po‐Kuei Wu, Paul Chih‐Hsueh Chen, Chia‐Wen Lee, Wei‐Ming Chen, Shih‐Chieh Hung

**Affiliations:** ^1^ Department of Orthopaedics and Traumatology, Taipei Veterans General Hospital, Taipei, Taiwan, Republic of China; ^2^ Department of Orthopaedics, Therapeutical and Research Center of Musculoskeletal Tumor, Taipei Veterans General Hospital, Taipei, Taiwan, Republic of China; ^3^ Institute of Traditional Medicine, School of Medicine, National Yang‐Ming University, Taipei, Taiwan, Republic of China; ^4^ Department of Pathology and Laboratory Medicine, Taipei Veterans General Hospital, Taipei, Taiwan, Republic of China; ^5^ Institute of Clinical Medicine, School of Medicine, National Yang‐Ming University, Taipei, Taiwan, Republic of China; ^6^ Department of Pharmacology, School of Medicine, National Yang‐Ming University, Taipei, Taiwan, Republic of China; ^7^ Medical Research, Taipei Veterans General Hospital, Taipei, Taiwan, Republic of China; ^8^ Institute of Biomedical Sciences, Academia Sinica, Taipei, Taiwan, Republic of China; ^9^ Integrative Stem Cell Center, China Medical University Hospital, Taichung, Taiwan, Republic of China; ^10^ Graduate Institute of Clinical Medical Science, China Medical University, Taichung, Taiwan, Republic of China

**Keywords:** Retinoblastoma (Rb) knockdown, c‐Myc, Mesenchymal stem cell, Osteosarcoma, Transformation

## Abstract

Osteosarcoma (OS) was a malignant tumor occurring with unknown etiology that made prevention and early diagnosis difficult. Mesenchymal stem cells (MSCs), which were found in bone marrow, were claimed to be a possible origin of OS but with little direct evidence. We aimed to characterize OS cells transformed from human MSCs (hMSCs) and identify their association with human primary OS cells and patient survival. Genetic modification with p53 or retinoblastoma (Rb) knockdown and c‐Myc or Ras overexpression was applied for hMSC transformation. Transformed cells were assayed for proliferation, differentiation, tumorigenecity, and gene expression profile. Only the combination of Rb knockdown and c‐Myc overexpression successfully transformed hMSCs derived from four individual donors, with increasing cell proliferation, decreasing cell senescence rate, and increasing ability to form colonies and spheres in serum‐free medium. These transformed cells lost the expression of certain surface markers, increased in osteogenic potential, and decreased in adipogenic potential. After injection in immunodeficient mice, these cells formed OS‐like tumors, as evidenced by radiographic analyses and immunohistochemistry of various OS markers. Microarray with cluster analysis revealed that these transformed cells have gene profiles more similar to patient‐derived primary OS cells than their normal MSC counterparts. Most importantly, comparison of OS patient tumor samples revealed that a combination of Rb loss and c‐Myc overexpression correlated with a decrease in patient survival. This study successfully transformed human MSCs to OS‐like cells by Rb knockdown and c‐Myc overexpression that may be a useful platform for further investigation of preventive and target therapy for human OS. Stem Cells Translational Medicine *2017;6:512–526*

**Significance Statement**

In the present study, transformation of human mesenchymal stem cells (MSCs) was achieved by the combination of retinoblastoma (Rb) knockdown and c‐Myc overexpression. After a robust evaluation with radiographic analyses, micro‐positron emission tomography/computed tomography, micro‐magnetic resonance imaging, and immunohistochemistry of various osteosarcoma (OS) markers, tumors were found to be formed by intraosseous and subcutaneous injection of these cells that were OS‐like tumors. Through the use of microarray with cluster analysis, these transformed cells were shown to have gene profiles more similar to those of patient‐derived primary OS cells than their normal MSC counterparts. More important, comparison of OS patient tumor samples revealed that a combination of Rb loss and c‐Myc overexpression correlated with a decrease in patient survival. These data strongly suggest that transformed human MSCs by Rb knockdown and c‐Myc overexpression are associated with clinical OS tissues and patient survival. Thus, the present study identifies a useful platform for further investigation of prevention and target therapy for human OS.

## Introduction

Osteosarcoma (OS), the most common bone cancer in young adults, occurs frequently in areas where there is rapid bone growth, such as the distal femur, the proximal tibia, and the proximal humerus 1. OS is locally aggressive and frequently metastasizes to the lung 2. Although a combination of surgery and chemotherapy results in long‐term survival in approximately 60%–70% of OS patients, those who do not respond to chemotherapy still have a poor prognosis 3. Because the etiology of OS is unknown, clarification of the mechanism for occurrence of OS is important for prevention and prediction.

Mesenchymal stem cells (MSCs), which were first discovered by Friedenstein et al. in 1974 4, are stromal cells and structural components of the bone marrow. They have the ability to differentiate to osteoblasts, adipocytes, and chondrocytes 5. Recent data have shown the linkage between MSCs and tumor formation. Circulating MSCs target microscopic tumors and contribute to a significant proportion of tumors 6. MSCs also promote tumor metastasis in a variety of cancers 7, 8. In addition, it has been reported that MSCs are the progenitor cells of certain sarcomas, such as OS 9. Usually, MSCs are quiescent and easily experience replicative senescence upon proliferation, but they proliferate and maintain differentiation potential if there is a stimulus or if there are gene mutations that cause transformation.

The transformation of MSCs has been achieved under the controlled expression or deletion of certain genes, such as by the expression of the *EWS‐FLI‐1* chimeric gene to induce Ewing sarcoma 10, the deletion of p53 to induce leiomyosarcoma 11, and the deletion of the retinoblastoma (*Rb*) gene to induce liposarcoma 12. Mouse models that allow inactive mutation of both p53 and Rb, which are responsible for cell cycle regulation and apoptosis 13, in the osteoblastic lineage, induce OS 14. Interestingly, the inactive mutation of p53 and Rb has been noted in the osteoblastic lineage of OS patients 15. Loss of either p53 or the *Rb* gene results in a high incidence of poor prognosis in OS patients 16. In addition to tumor suppressor genes, oncogenes, such as Ras, Raf 17, and c‐Myc 18, play a major role in the oncogenic transformation of normal cells. c‐Myc is one of the genes that is required for somatic cell reprogramming into the pluripotent state (induced pluripotent‐like stem cell) 19. Notably, more than 10% OS patients have c‐Myc amplification 20, 21.

Because most studies of MSC transformation use rodent MSCs 9, 12, 14, 22, 23, 24, which innately have more unstable genomes 25 than human MSCs during ex vivo expansion 26, 27, the tumorigenic ability of human MSCs is still unclear. This study transforms human MSCs via genetic modification of several genes, including the knockdown of p53 and Rb and the overexpression of c‐Myc and Ras. Human MSCs resist immortalization by single‐gene modification or some other combinations. In our study, the combination of Rb knockdown and c‐Myc overexpression immortalizes human MSCs. By using this combination, the in vitro and in vivo OS models were derived from human MSCs. By comparing the transcriptomes, it is demonstrated that human MSC‐derived OS cell lines are more similar to the primary OS cell lines of OS patients than their parental human MSCs and the corresponding primary MSCs of OS patients. These transformed MSC lines will be useful for further therapeutic investigation.

## Materials and Methods

### Cells and Culture Conditions

These studies were approved by the Institutional Review Board of Taipei Veterans General Hospital, with informed consent obtained from healthy donors who provided bone marrow aspirates and from OS patients who provided tumor specimens and normal bone marrow aspirates. The primary MSCs were isolated from bone marrow aspirates of donors who received traumatic surgery and signed the consent forms. After centrifugation with 1.077 Ficoll‐Hypaque (Sigma‐Aldrich, St. Louis, MO, http://www.sigmaaldrich.com) for 10 minutes at 550*g*, mononuclear cells located in the middle layer were isolated and cultured onto 10‐cm dishes containing incomplete medium (α‐MEM; Thermo Fisher Scientific Life Sciences, Oakwood Village, OH, https://www.thermofisher.com) supplemented with 10% fetal bovine serum (FBS; Thermo Fisher), 100 units/ml penicillin/streptomycin (Thermo Fisher), and 2 mM L‐glutamine (Sigma‐Aldrich). After 1 day of culture, the unattached cells were removed, and the rest cells were continuously cultured as MSC lines. The MSCs used for transformation were among passages 3–5, and those used for other experiments were less than passage 8. After passage 10, the MSCs underwent senescent change 28. The MG63 human osteosarcoma cell line (CRL‐1427), characterized as an osteoblastic type, was purchased from ATCC (Rockville, MD, http://www.atcc.org) and cultured in high‐glucose Dulbecco's modified Eagle's medium (DMEM) with 10% FBS. Cells were subcultured by 0.25% trypsin (Thermo Fisher) digestion. With the capacity for osteogenesis and colony formation 29, 30, MG63 was used as the positive control in osteogenic and tumorigenic assays. Spheroid formation assay was performed in tumor sphere medium (TSM; serum‐free DMEM/F12 [Thermo Fisher] containing N2‐supplement [Thermo Fisher], human epidermal growth factor [20 ng/ml; Peprotech, Rocky Hill, NJ, http://www.peprotech.com], and basic fibroblast growth factor [10 ng/ml, Peprotech]), and the sphere numbers were counted at day 14.

### Transfection and Lentiviral‐Mediated Transduction

The expression plasmids and the bacteria clone and lentiviral vectors carrying p53 (TRCN0000003755 and TRCN0000003756), Rb (TRCN0000010417 and TRCN0000040167), and β‐catenin (TRCN0000003845) short hairpin RNAs were provided by the National Science Council RNAi core facility, Academia Sinica, Taiwan. Subconfluent cells were infected with lentivirus (1.3 × 10^7^ refractive index unit/ml, infected with 3 multiplicities of infection) in the presence of 8 μg/ml polybrene (Sigma‐Aldrich). At 24 hours after infection, medium was removed and replaced with fresh growth medium containing puromycin (1 μg/ml) to select for infected cells. Overexpression of Ras or c‐Myc in MSCs was infected with retrovirus carrying Ras or c‐Myc that was kindly provided by Shih‐Hwa Chiou (National Yang‐Ming University) 31.

### Short Tandem Repeat Profiling

The short tandem repeat (STR) profiling analysis was performed by the team of Dau‐Ming Niu (Director of Research and treatment Center of Rare Disease and Genetic Consultant Centerin, Taipei Veterans General Hospital). In brief, the genomic DNA of MSCs and silence of Rb‐overexpression of c‐Myc (SiRb‐OeMyc) cells were extracted by using the High Pure polymerase chain reaction (PCR) Template Preparation Kit (Roche, Mannheim, Germany, http://www.roche.com). A total of 2 ng of target DNA was performed with the AmpF/STR Identifiler PCR Amplification kit (Thermo Fisher) following the manufacturer's instructions. The data were analyzed by the ABI Prism 310 Genetic Analyzer (Thermo Fisher) 32.

### Calculation of Cumulative Population Doublings

Cells were initially seeded at 1 × 10^3^ cells per cm^2^ in complete medium with continuous subculture for every 4 days, and the cell number was counted by the Trypan blue exclusion method (Sigma‐Aldrich) for a total of five passages. Cumulative population doublings (CPDs) were calculated by using the formula: CPD = [log_10_ (*N*_H_) − log_10_(*N*_1_)]/log_10_^2^, where *N*_H_ is the harvested cell number and *N*_1_ is the plated cell number. The generation time was calculated as: [log_10_^2^ × Δ*t*]/[log_10_(*N*_H_) − log_10_(*N*_1_)], where Δ*t* is the time between passages 11.

### In Vitro Colony Formation Assay

To evaluate anchorage‐independent growth, 5,000 cells were resuspended in 0.33% agarose (Sigma‐Aldrich) in growth medium and plated on a solidified bed of 3% agarose in growth medium in six‐well plates. Plates containing each kind of cells were fixed and stained with 0.005% crystal violet (Sigma‐Aldrich) after 14 days of growth. Bright‐field images of cell colonies were taken by using a ×4 objective. The number of colonies with a diameter greater than 200 μm was counted per plate.

### Flow Cytometry for Surface Marker Analysis

The expression levels of MSC surface markers were determined by flow cytometry assay. Briefly, suspension cells were incubated for 30 minutes at 4°C with fluorescein isothiocyanate‐ or phycoerythrin‐conjugated monoclonal antibodies to human CD markers in 50 µl of washing buffer (phosphate‐buffered saline [PBS] with 2% FBS). After incubation, cells with bound antibodies were washed twice with washing buffer and fixed in 1% paraformaldehyde (in PBS). Cells were analyzed using a FACScan flow cytometer running CellQuest software (BD Biosciences, San Jose, CA, http://www.bdbiosciences.com).

### Tetrazolium‐Based Colorimetric Assay [3‐(4,5‐dimethylthiazol‐2‐yl)‐2,5‐diphenyltetrazolium bromide)

Cell growth was determined with the 3‐(4,5‐dimethylthiazol‐2‐yl)‐2,5‐diphenyltetrazolium bromide (MTT) cell proliferation assay kit (Sigma‐Aldrich). For this, 5 × 10^3^ cells per well were seeded onto 96‐well plates in 100 μl of culture medium. After incubation with the different treatments, cells were exposed to the MTT dye (5 mg/mL) and incubated at 37°C for 3 hours. The resulting formazan crystals were solubilized with dimethyl sulfoxide, and the absorbance was measured at 570 nm with a multiscan autoreader (M1000 PRO, Tecan, Männedorf, Switzerland, http://www.tecan.com). The results are presented as mean ± SD of fold change relative to day 0 (*n* = 3).

### Osteogenesis and Adipogenesis Induction

The in vitro differentiation toward an osteoblast's phenotype was induced in osteogenic induction medium (OIM; complete medium supplemented with 50 mg/ml ascorbate‐2 phosphate, 10^−8^ M dexamethasone, and 10 mM β‐glycerophosphate [all from Sigma‐Aldrich]] for 2 weeks, followed by Alizarin Red S (Sigma‐Aldrich) staining and quantification by measurement of optical density at 550 nm (OD_550_). Adipocytic differentiation was induced by adipogenic induction medium (complete medium supplemented with 50 mg/ml ascorbate‐2 phosphate, 10^−7^ M dexamethasone, 50 mM indomethacin, 0.45 mM 3‐isobutyl‐1‐methyl‐xanthine, and 10 mg/ml insulin [all from Sigma‐Aldrich]) for at least 2 weeks, followed by Oil Red O (Sigma‐Aldrich) staining and quantification by measurement of OD_510_.

### In Vivo Xenograft Tumorigenesis Assay

Study protocols involving animals were approved by the Institutional Animal Committee of Taipei Veterans General Hospital (Institutional Animal Care and Use Committee No. 2011‐077). Immunodeficient NU‐Foxn1nu mice (BioLasco, Taiwan Co., Ltd., Taipei, Taiwan, Republic of China, http://www.biolasco.com.tw), were housed as a cloned colony under specific pathogen‐free conditions in the Taipei Veterans General Hospital Animal Facility. The mice at 6–8 weeks old received an intratibial injection of 1 × 10^7^ cells. Tumor size and body weight were measured every week, and tumor volume was determined as length × width^2^/2. At 4–8 weeks of tumor injection, mice were anesthetized for radiological analysis of the bone with the x‐ray image (AXR minishot, Associated X‐Ray, East Haven, CT, http://www.associatedxray.com) by using a 3‐second exposure at 30 kV. After this, the animals were sacrificed.

### Micro‐Positron Emission Tomography/Computed Tomography

Micro‐positron emission tomography (micro‐PET)/computed tomography (CT) for dynamic study was conducted on mice before sacrifice. Briefly, the mice were injected with 0.3 mCi [^18^F]‐FDG via tail vein followed by micro‐PET R4 scanner (Concorde MicroSystems, Knoxville, TN, http://microscopy.wisc.edu/equipment/siemens-inveon-hybrid-micro-petct-scanner) (with the energy window being 350–650 keV and timing window being 6 ns). Dynamic sinograms were produced with 12 × 10‐, 6 × 30‐, 5 × 300‐, 3 × 600‐, and 4 × 900‐second frames. The images were reconstructed by the Fourier rebinning algorithm and two‐dimensional filtered back projection using a ramp filter with cutoff at Nyquist. All these processes were carried out by MicroPET Manager (version 2.3.3.6) under the instructions of the manufacturer. The PET images were analyzed by using ASIPro VM6.3.3.1 software (Concorde MicroSystems). A cylinder calibration method was used to convert the image units from counts per second per voxel to nCi per cm^3^. Finally, the CT scan was performed by the same machine.

### Immunohistochemistry

Bone tumor markers were analyzed by immunohistochemistry. Tissue sections from formalin‐fixed and paraffin‐embedded tissue, 5 μm thick, were stained with hematoxylin‐eosin (Sigma‐Aldrich) for histopathological analysis. For immunohistochemistry, the dewaxed and rehydrated sections were retrieved by sodium citrate buffer (10 mM with 0.05% Tween 20, pH 6.0; Sigma‐Aldrich) in a 95°C water bath for 20 minutes and blocked by 3% H_2_O_2_ after the slides were cooled down. Antibodies against tumor markers, such as human specific FLI‐1 (mouse IgG2a anti‐human, 1:200; BD Biosciences, catalog no. 554267), S100 (rabbit polyclonal anti‐S100, 1:300; IS504, Dako, Glostrup, Denmark, http://www.dako.com), desmin (mouse IgG1, κ, 1:120; Dako, IS606), α‐smooth muscle actin (α‐SMA; mouse anti‐human α‐SMA, clone 1A4,1:100; Dako, M0851), alkaline phosphatase (ALP; mouse IgG2b, clone 4H1,1:200; ab54778; Abcam, Cambridge, MA, http://www.abcam.com), osteonectin (mouse IgG1, clone 5031, 1:10; catalog no. MA1‐43027, Applied Biosystems, Singapore, http://www.appliedbiosystems.com), and osteocalcin (rabbit polyclonal, FL‐100, 1:50; catalog no. sc‐30044, Santa Cruz Biotechnology Inc., Santa Cruz, CA, http://www.scbt.com) were applied to specimens. The Dako LSAB kit containing mouse anti‐human secondary antibody was used for detection. The CD99 (rabbit polyclonal IgG, 1:100; GeneTex, Irvine, CA, http://www.genetex.com) biomarker that is also found in OS 33 also was stained on the SiRb‐OeMyc in vivo tumor section. All of the immunohistochemistry data, including positive (from patient tumor sections) and negative controls (without first antibodies), have been examined by a clinical pathologist (P.C.‐H.C.).

### Western Blotting

Cell extracts were prepared with mammalian protein extraction reagent (M‐PER; Thermo Fisher) plus protease inhibitor cocktail (Halt), and protein concentrations were determined by using the bicinchoninic acid assay (Thermo Fisher). Aliquots of protein lysates were separated on SDS‐10% polyacrylamide gels and transferred to polyvinylidene difluoride membrane filters, which were blocked with 5% blotting grade milk (Bio‐Rad, Hercules, CA, http://www.bio-rad.com) in TBST (20 mM Tris‐HCl [pH 7.6], 137 mM NaCl, 1% Tween 20). Membranes were then probed with the indicated primary antibodies, reacted with corresponding secondary antibodies, and detected by using a chemiluminescence assay (EMD Millipore, Billerica, MA, http://www.emdmillipore.com). Membranes were exposed to x‐ray film to visualize the bands (GE Healthcare Life Sciences, Piscataway, NJ, http://www.gelifesciences.com). The primary antibodies included anti‐p53, anti‐Rb, anti‐Oct4, anti‐SOX‐2, anti‐Nanog, anti‐c‐Myc, anti‐β‐catenin, and anti‐β‐actin (1:1,000; Cell Signaling Technology, Beverly, MA, http://www.cellsignal.com). The secondary antibodies included horseradish peroxidase‐conjugated donkey anti‐rabbit or anti‐mouse antibodies (1:2,000; GeneTex). The quantification of each protein expression level was normalized by internal control α‐tubulin, and the expression level of parental MSC was referred to as 1.

### High‐Throughput Gene Analysis by Microarray

The total RNA was extracted from cells by using TRIzol reagent (Thermo Fisher) according to the manufacturer's specifications. The total RNA was reverse‐transcribed with Superscript II RNase H‐reverse transcriptase (Thermo Fisher), followed by Illumina microarray analysis of human HT‐12 array. The raw data are available from the Gene Expression Omnibus database under accession number GSE56001. The results from microarray were analyzed by using GeneSpring software (GeneSpring X, Agilent Technologies, Santa Clara, CA, http://www.agilent.com). The array data were further confirmed by quantitative real‐time PCR with the SYBR system (Protech Technology, Taipei, Taiwan, Republic of China, http://www.protech-bio.com). The statistical analysis of gene array was analyzed by one‐way analysis of variance (ANOVA), followed by the Student‐Newman‐Keuls method, which is a stepwise multiple comparisons procedure obtained by GeneSpring software.

### Plasmids and Reporter Gene Assays

TOPFLASH and FOPFLASH were purchased from Thermo Fisher. For the reporter analyses, 5,000 cells were seeded on 96‐well plates for transfection. A total of 0.2 μg of each DNA was transfected, including a TK‐renilla luciferase vector (Promega, Madison, WI, http://www.promega.com). The luciferase activities were measured by using the Dual‐Luciferase Assay System (Promega).

### Tissue Array Immunohistochemistry

The cohort study was based on OS tissue samples collected at the Department of Orthopedics and Traumatology, Taipei Veterans General Hospital, from 1992 to 2011. The cohort was followed for mortality until 2014. For cancer deaths, the underlying causes of death were determined by death certificates and selected according to the version of the International Classification of Diseases codes at the time of death. Tissue arrays collected from 111 OS patients were analyzed by immunohistochemistry for Rb and c‐Myc expression patterns. After the exclusion of missing samples, lost follow‐up patients, and duplicate collections, a total of 72 tumor spots from each enrolled subject were applied for this assay. The antibodies for immunohistochemistry were anti‐human c‐Myc (rabbit polyclonal, clone D3N8F, 1:50; Cell Signaling) and anti‐human Rb1 (mouse monoclonal, clone 4H1, 1:50; Cell Signaling).

### Statistical Methods

The data were expressed as mean ± SD. Independent *t* test was performed for comparison of data from two independent samples. More than two groups were compared by one‐way ANOVA with Bonferroni post hoc test, and more than two groups with different time points were analyzed by two‐way ANOVA with post hoc test. A *p* value of < .05 was considered statistically significant and labeled with ∗, *p* < .05; ∗∗, *p* < .01; ∗∗∗, *p* < .005.

## Results

### The Combination of Rb Knockdown and c‐Myc Overexpression Transformed MSCs

**Figure 1:**
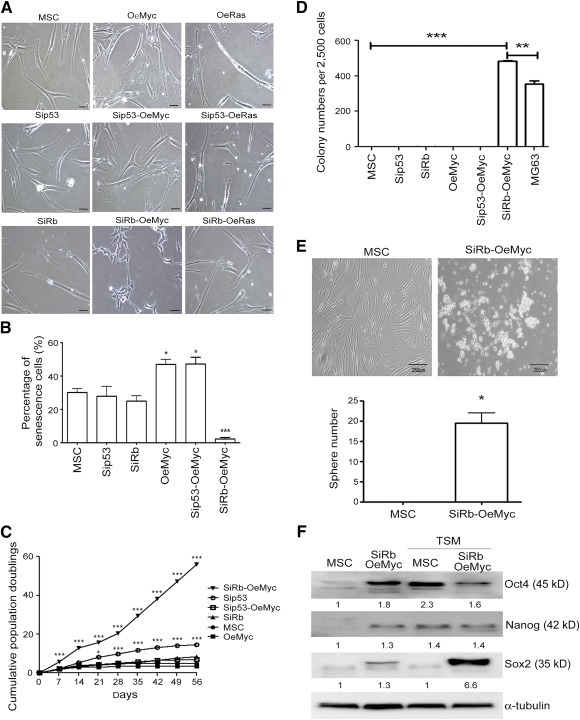
Combination of Rb knockdown and c‐Myc overexpression transforms MSCs. **(A):** Parental MSCs or MSCs with genetic modification were grown in complete medium. Morphological changes were observed after genetic modification. **(B):** β‐galactosidase staining for analyzing senescence showed abundant senescent cells in OeMyc and Sip53‐OeMyc. Percentage of senescent cells in MSCs with different genetic modification. ∗, *p* < .05; ∗∗∗, *p* < .005 compared with MSC. **(C):** Cells were continuously subcultured in complete medium, and cumulative population doublings were calculated. **(D):** Cells were assayed for soft agar colony formation ability. **(E):** Cells were assayed for sphere formation in TSM. **(F):** Cells in TSM were subjected to Western blotting analysis for pluripotency gene expression. ∗, *p* < .05; ∗∗, *p* < .01; ∗∗∗, *p* < .005 compared with MSC. Bar = 500 μm. Abbreviations: MSC, mesenchymal stem cell; OeMyc, overexpression of c‐Myc; Rb, retinoblastoma; Sip53, silence of p53; SiRb, silence of retinoblastoma; TSM, tumor sphere medium.

Human MSCs were genetically manipulated to study the genetic alterations required to reprogram them into tumor cells. Knockdown of p53 and Rb, the most common tumor suppressers, and overexpression of c‐Myc and Ras, the most common oncogenes, were undertaken in human MSCs by using lentiviral transduction. Efficient knockdown or overexpression for each gene was confirmed by the obvious change in protein and in mRNA levels (supplemental online Fig. 1). Single knockdown of p53 (Si53) or Rb (SiRb) or double knockdown of p53 and Rb (Sip53 and SiRb) resulted in no change in the morphology or the growth rate (Fig. 1A), but the overexpression of c‐Myc (OeMyc) or Ras (OeRas) (data not shown) evoked oncogenic stress‐induced senescence (Fig. 1B). A combination of p53 knockdown and c‐Myc overexpression (Sip53‐OeMyc) also induced cell senescence and a termination of cell growth (Fig. 1B). Interestingly, Rb knockdown combined with c‐Myc overexpression (SiRb‐OeMyc), but not Ras overexpression (SiRb‐OeRas; data not shown), resulted in a slim and short cell shape, a significant decrease in cell size (Fig. 1A), the inhibition of senescence (Fig. 1B), and increased cell growth (Fig. 1C). The in vitro soft agar assay also showed that cells with SiRb‐OeMyc have a greater ability to form colonies than different single or combined gene mutations, and the ability to form colonies is even greater than that of MG63, an OS cell line as positive control 29, 30 (Fig. 1D). To confirm the transformation ability of genetic modification with SiRb‐OeMyc, MSCs from three other donors were also applied for this study. The genetically modified MSC strains also showed similar morphology change and an increase in colony formation ability on soft agar assay (supplemental online Fig. 2). It was also found that cells with SiRb‐OeMyc increased the ability to form spheres in TSM, the in vitro culture medium for cancer stem cells 34 (Fig. 1E) and expressed pluripotency genes, such as Oct4, Nanog, and Sox2, both before and after culture in TSM (Fig. 1F). The comparison of 15 STR loci between parental MSCs and the corresponding SiRb‐OeMyc cells revealed that these two cells showed high similarity, excluding the possibility of contamination (Table 1). Together, these data suggest that SiRb‐OeMyc, rather than Sip53, SiRb, OeMyc, or OeRas, was required for the oncogenic transformation of human MSCs into tumor cells.

**Table 1:** Short tandem repeat profiling of parental MSCs and SiRb‐OeMyc cells

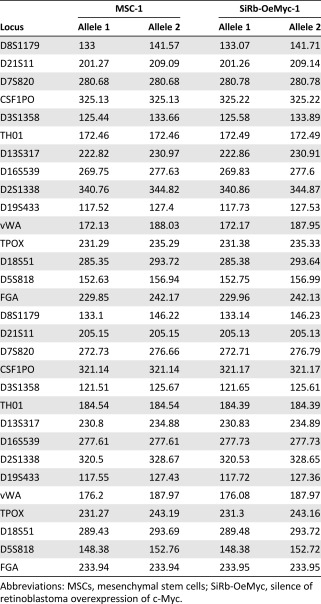

### A Combination of Rb Knockdown and c‐Myc Overexpression Induced Phenotypic Changes in MSCs

**Figure 2:**
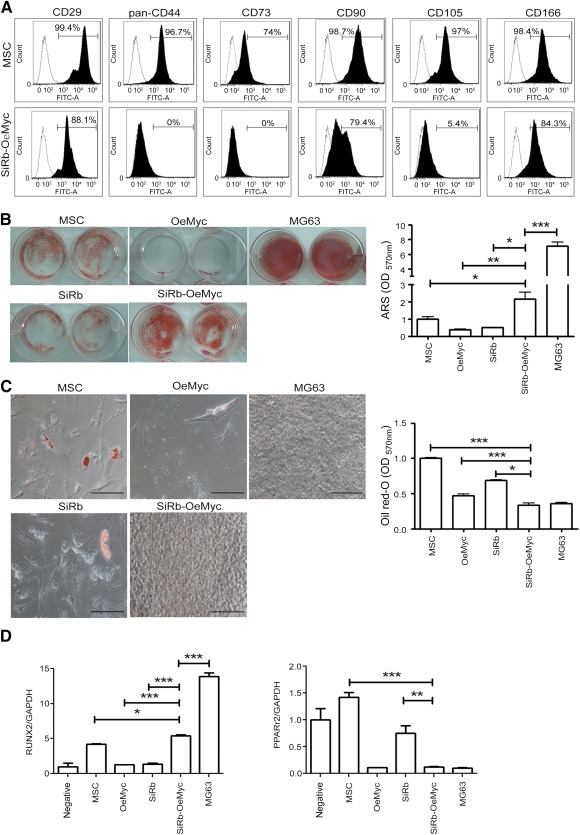
Combination of retinoblastoma (Rb) knockdown and c‐Myc overexpression caused phenotypic changes. **(A):** Flow cytometric analysis for cell surface markers in parental MSCs and MSCs with SiRb and c‐Myc overexpression (SiRb‐OeMyc). **(B** left**):** Cells were induced for osteogenesis in osteogenic induction medium for 2 weeks, followed by ARS staining. **(B** right**):** Stained dye was extracted, and OD values were measured. **(C** left**):** Cells were induced for adipogenesis in adipogenic induction medium for 2 weeks, followed by Oil Red O staining. **(C** right**):** Stained dye was extracted, and OD values were measured. Bars = 100 μm. The gene expression levels of RUNX2 at 7 days of osteogenic induction and PPARγ2 at 7 days of adipogenic induction were analyzed by quantitative reverse‐transcriptase polymerase chain reaction assay. **(D):** Compared with parental MSCs, SiRb‐OeMyc cells significantly increased in RUNX2 mRNA level (left), whereas they decreased in PPARγ2 mRNA level (right). ∗, *p* < .05; ∗∗, *p* < .01; ∗∗∗, *p* < .005 compared with MSC. Abbreviations: ARS, Alizarin Red S; GAPDH, glyceraldehyde‐3‐phosphate dehydrogenase; MSC, mesenchymal stem cell; OD, optical density; OeMyc, overexpression of c‐Myc; PPARγ2, peroxisome proliferator‐activated receptor γ2; Rb, retinoblastoma; RUNX2, runt‐related gene 2; SiRb, silence of Rb.

In order to determine the phenotypic changes that occur upon transformation of MSCs into tumor cells, the surface protein profiles and differentiation potential of the MSCs, without or with either or both Rb knockdown and c‐Myc overexpression, were measured. Flow cytometry showed that, after 4 days of continuous culture, more than 97% of the MSCs were positive for putative MSC CD markers (Fig. 2A), and several putative CD surface markers, such as CD29, CD44, CD73, CD90, CD105, and CD166, were decreased or lost in SiRb‐OeMyc cells (Fig. 2A). When osteogenic differentiation was induced in a defined medium, SiRb‐OeMyc cells showed an increase in osteogenic differentiation (2.2‐ ± 0.65‐fold) compared with parental cells (normalized as 1), without, or with Rb knockdown (0.5‐ ± 0.01‐fold), or c‐Myc overexpression (0.4‐ ± 0.02‐fold) (Fig. 2B). However, the degree of osteogenic differentiation was close to, but still less than, that of MG63 (7.1‐ ± 0.95‐fold) (Fig. 2B). In contrast, SiRb‐OeMyc cells showed decreased adipogenic differentiation, compared with parental cells, without, or with Rb knockdown, or c‐Myc overexpression, when adipogenic differentiation was induced in a defined medium (Fig. 2C; data not shown). The gene expression levels of runt‐related gene 2 (RUNX2) at 7 days of osteogenesis induction and peroxisome proliferator‐activated receptor γ2 (PPARγ2) at 7 days of adipogenesis induction were analyzed by quantitative reverse‐transcriptase PCR assay. Compared with parental MSCs, SiRb‐OeMyc cells significantly increased in the RUNX2 mRNA level (Fig. 2D left), whereas they decreased in the PPARγ2 mRNA level (Fig. 2D right). The adipogenic potential of SiRb‐OeMyc cells was also not as good as that of MG63, which suggests a loss of adipogenic potential. These data suggest that a combination of Rb knockdown and c‐Myc overexpression transformed human MSCs into tumor cells and induced phenotypic changes, including the loss of MSC CD surface markers, an increase in osteogenic potential, and a decrease in adipogenic potential.

### The Formation of Intraosseous Tumors by SiRb‐OeMyc Cells in Nude Mice

**Figure 3:**
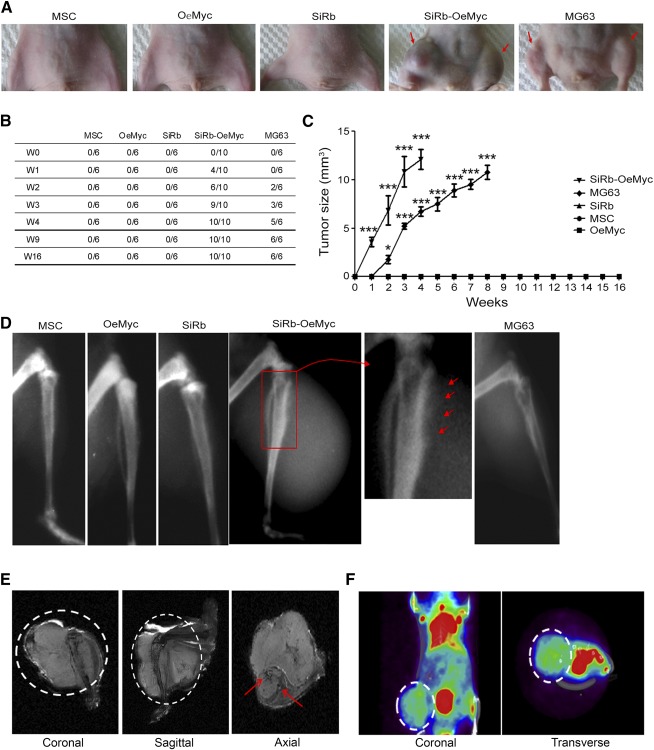
Intraosseous tumor formation by SiRb‐OeMyc cells in nude mice. **(A):** After intraosseous injection, only SiRb‐OeMyc and MG63 cells formed tumor (arrows) at 4 weeks. **(B):** The ratio of tumor formation for each injection at the indicated time period after injection is shown. **(C):** Tumor growth curve after intraosseous injection. ∗, *p* < .05; ∗∗∗, *p* < .005. **(D):** Representative x‐ray plain films show tumor formation in SiRb‐OeMyc and MG63. The magnified rectangle shows sunburst features (arrows). **(E):** Representative magnetic resonance imaging images of intraosseous tumor formed by SiRb‐OeMyc. Dotted lines in coronal and sagittal views are tumor ranges and arrows in axial view show the location of tibia and fibula. **(F):** Representative micro‐positron emission tomography/computed tomography images for tumor location outlined by dotted lines. Abbreviations: MSC, mesenchymal stem cell; OeMyc, overexpression of c‐Myc; SiRb, silence of retinoblastoma; W, week.

In order to determine whether in vitro oncogenic transformation is associated with in vivo oncogenic transformation, genetically altered MSC cells were injected into the tibiae (the orthotopic site) of immunodeficient mice, for an in vivo tumorigenecity assay. Some of the mice that were injected with 1 × 10^7^ SiRb‐OeMyc cells began to form tumors at 1 week after injection, but mice injected with 1 × 10^7^ MG63 did not begin to form tumors until 2–3 weeks after injection (Fig. 3A). All 10 mice that underwent intratibial injection of SiRb‐OeMyc cells showed tumor formation 4 weeks after injection, but those that received other genetically altered cells showed no tumor formation 16 weeks after injection (Fig. 3B). The size of the tumors formed by SiRb‐OeMyc were also significantly greater than that of tumors formed by MG63, in the early weeks after injection (Fig. 3A, 3C). Radiographic analysis with x‐ray also showed periosteal reaction and the appearance of onion skin in the tibiae injected with SiRb‐OeMyc (Fig. 3D, arrows). The MRI image (Fig. 3E) and micro‐PET/CT (Fig. 3F) also showed enhanced signals in the tumor cell injection site. These data suggested that SiRb‐OeMyc, rather than Sip53, SiRb, OeMyc, or OeRas, induced in vivo oncogenic transformation of human MSCs in the orthotopic site. The transformed MSCs from the other three individuals could also form tumors in nude mice, further confirming the tumorigenic ability of Rb silencing and c‐Myc overexpression in human MSCs (supplemental online Fig. 3).

### Tumors Formed by SiRb‐OeMyc Have OS Features

**Figure 4:**
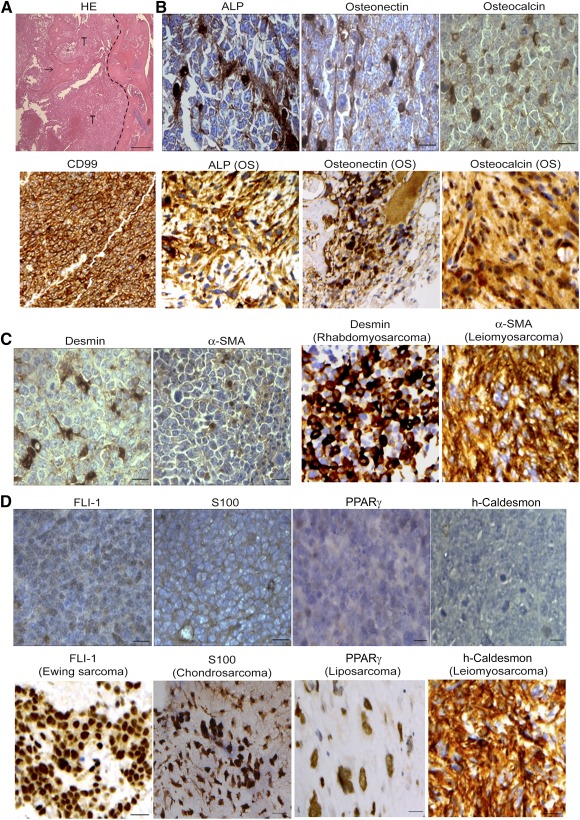
Tumors formed by retinoblastoma SiRb‐OeMyc express OS features. **(A):** HE staining of intraosseous tumor formed by SiRb‐OeMyc showed new bone formation (arrow) within tumor. Dotted line indicates the tumor margin. **(B–D):** Immunohistochemistry shows tumors formed by SiRb‐OeMyc are positive for OS markers such as CD99, ALP, osteonectin, and osteocalcin **(B**, upper panels; brown**)**; and slightly positive for sarcoma markers such as desmin and α‐SMA **(C**, left panels; brown**)**; but negative for markers of Ewing sarcoma (FLI‐1), chondrosarcoma (S100), liposarcoma (PPARγ), and leiomyosarcoma (h‐caldesmon) **(D)**. Positive controls of immunohistochemistry are from human pathological sections of OS **(B**, lower panels**)**, rhabdomyosarcoma, leiomyosarcoma **(C**, right panels**)**, Ewing sarcoma, chondrosarcoma, liposarcoma, and leiomyosarcoma **(D**, upper panels**)**. Bars = 100 μm. Abbreviations: ALP, alkaline phosphatase; HE, hematoxylin and eosin; OS, osteosarcoma; PPARγ, peroxisome proliferator‐activated receptor γ; α‐SMA, α‐smooth muscle actin; T, tumor.

In order to determine whether the tumors formed by SiRb‐OeMyc are OS or other types of musculoskeletal malignancies, we used the markers for tumors formed by SiRb‐OeMyc, such as OS, Ewing sarcoma, chondrosarcoma, liposarcoma, and leiomyosarcoma. The HE stain showed that the tumors formed by SiRb‐OeMyc have osteoid tissues (Fig. 4A, arrow). Immunohistochemistry further showed that the tumors were positive for several putative OS markers, such as CD99 33, ALP 35, osteonectin 36, and osteocalcin 37 (Fig. 4B), and partially positive for sarcoma markers, such as desmin 38 and α‐SMA 38 (Fig. 4C). The markers of other sarcoma types, such as Ewing sarcoma marker, FLI‐1 38, chondrosarcoma or neurogenic sarcoma marker, S100 38, liposarcoma marker, PPARγ 39, and leiomyosarcoma marker h‐caldesmon 38, tested negative in tumors formed by SiRb‐OeMyc (Fig. 4D). These data suggested that SiRb‐OeMyc cells form OS‐like tumors in the orthotopic site of immunodeficient mice (supplemental online Fig. 3D). In addition, human nuclei staining (anti‐human nuclei antibody, clone 235‐1, EMD Millipore, catalog no. MAB1281) was performed to confirm that the xenograft tumors had originated from human cells rather than host cells. The tumors formed by SiRb‐OeMyc cells in tibia were significantly positive for human nuclei (dark brown spots), whereas neighboring nontumor parts were negative for human nuclei (blue spots). Notably, some human nuclei‐negative cells scattered among the tumors, indicating that there are some host cells in the xenograft tumors (supplemental online Fig. 4H, left).

### SiRb‐OeMyc Cell Xenografts Were Tumorigenic and Capable of Metastatic Secondary Tumors

A subcutaneous (s.c.) xenograft assay determined the tumorigenic and metastatic capacity of SiRb‐OeMyc cells. Immunodeficient mice were injected with the same number of SiRb‐OeMyc cells, and the rate of tumor formation was measured up to 16 weeks after injection (supplemental online Fig. 4A, 4B). At 4 weeks after injection, tumors formed in all of the s.c. injection sites. The s.c. tumors also had osteoid features and were positive for ALP, osteonectin, and osteocalcin (supplemental online Fig. 4C). In addition to local tumors, several lung and liver metastasis nodules were detected after animal sacrifice at 4 weeks after cell injection (supplemental online Fig. 4D). The tumor cells isolated from the primary xenograft tumor in tibiae still maintained the ability to form secondary tumors, after intratibial injection (supplemental online Fig. 4E). The MRI and micro‐PET/CT images of the secondary tumors also showed enhanced signals in tumor areas (supplemental online Fig. 4F, 4G). These data showed the ability for metastasis and secondary tumorigenesis of SiRb‐OeMyc cells.

### The SiRb‐OeMyc Cells Had a Similar Gene Profile to Human Primary OS Cells

**Figure 5:**
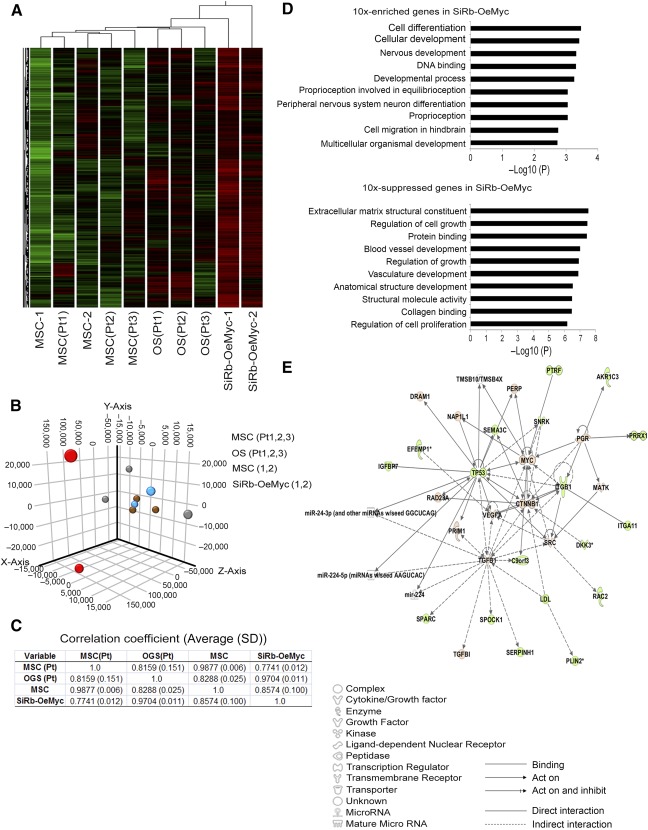
The SiRb‐OeMyc cells have similar gene profile as human primary OS cells. **(A):** Analyzing microarray data using cluster analysis. Heat map of genes with significant induction (red) or repression (green) showed the location of SiRb‐OeMyc behind three primary OS cells isolated from OS patients, whereas parental MSCs were close to primary MSCs from OS patients. **(B):** The principal component analysis shows both SiRb‐OeMyc and primary OS cells located far away from parental and patients’ derived MSCs, and scattered at different locations reflecting the diversity of tumor cells. **(C):** The correlation coefficient between SiRb‐OeMyc cells and primary OS cells is nearly 1, indicating the similarity of SiRb‐OeMyc cells and OS cells. **(D):** Gene Ontology analysis for 10‐fold upregulated or downregulated genes compared between SiRb‐OeMyc and parental MSCs. **(E):** Ingenuity pathway analysis for 10‐fold difference genes. The upregulated genes are marked as red and downregulated genes are marked as green, and no‐difference genes are shown as white. Abbreviations: MSC, mesenchymal stem cell; MSC‐1, parental MSC 1; OeMyc, overexpression of c‐Myc; OGS, osteogenic sarcoma; OS, osteosarcoma; Pt, OS patient; SiRb, silence of retinoblastoma.

In order to determine the changes in the gene expression profile in SiRb‐OeMyc cells, gene expression profiles of SiRb‐OeMyc cells were analyzed by using microarray transcriptomes and compared with those of their parental human MSCs and three primary OS cells and corresponding primary MSCs isolated from individual OS patients. Clustering of the gene profiles showed that the expression patterns of SiRb‐OeMyc cells were away from their parental MSCs and located behind three OS cells (Fig. 5A) and much different from other types of bone tumors (supplemental online Fig. 6). The principal component analysis (Fig. 5B) also revealed that parental MSCs and MSCs from patients were clustering together with correlation coefficient (CC) = 0.9877 (Fig. 5C), whereas both SiRb‐OeMyc and primary OS located far away from parental and patients’ derived MSCs and scattered at different locations with CC = 0.9704 (Fig. 5C), reflecting the diversity of tumor cells. Compared with parental MSCs, Gene Ontology (GO) analysis showed a 10‐fold upregulation of genes that were associated with cell differentiation, cell development, nervous system development, and DNA binding in SiRb‐OeMyc cells, and a 10‐fold downregulation of genes that were associated with extracellular matrix structural constituent, regulation of cell growth, and protein binding in SiRb‐OeMyc cells (Fig. 5D). The Ingenuity pathway analysis, a signaling network, showed a link between the 10‐fold difference in genes in SiRb‐OeMyc and parental MSCs (Fig. 5E). It was obvious from the network that there were two major signal transduction nodes: (a) affiliation with the induction of c‐Myc and (b) affiliation with the induction of p53. Minor signaling nodes appear to be routed through vascular endothelial growth factor A (VEGFA), β‐catenin gene, and transforming growth factor‐β 1 (TGF‐β1). To test the usefulness of the microarray data and their associated functional gene analysis, we first demonstrated that SiRb‐OeMyc cells increased mRNA expression and protein levels of β‐catenin (supplemental online Fig. 5A, 5B). Moreover, reporter gene analyses revealed that SiRb‐OeMyc cells increased in the activity of a TCF4 reporter plasmid, TOPFLASH, but not in the activity of the control FOPFLASH reporter containing mutated TBE sites (supplemental online Fig. 5C). We further demonstrated that the increase of β‐catenin level by combined Rb knockdown and c‐Myc overexpression was because of increased phosphorylation of glycogen synthase kinase‐3β (GSK3β), a kinase that negatively regulates β‐catenin 40 (supplemental online Fig. 5D). More importantly, knockdown of β‐catenin (supplemental online Fig. 5D) significantly suppressed cell growth (supplemental online Fig. 5E) and soft agar colony formation (supplemental online Fig. 5F). These data together suggested the involvement of the Wnt‐β‐catenin pathway in SiRb‐OeMyc‐mediated circumvention of senescence and induction of tumorigenesis in MSCs.

### The Expression Pattern of Rb Negative and c‐Myc Positive Correlated With Poor Prognosis in OS Patients

**Figure 6:**
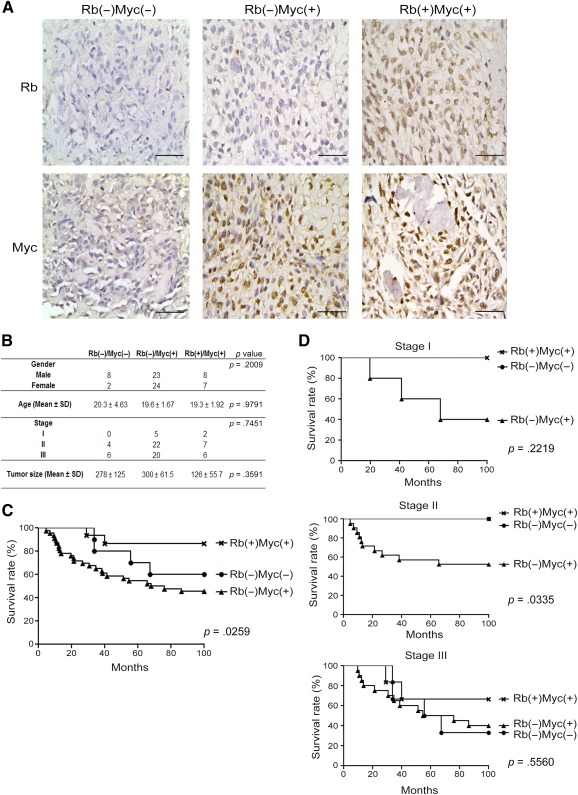
The expression level of Rb and c‐Myc correlated of human osteosarcoma (OS) survival rate. **(A):** The immunohistochemistry of Rb and c‐Myc in 72 human OS patients’ tissue array showed three expression patterns, Rb(−)Myc(−), Rb(−)Myc(+), and Rb(+)Myc(+). **(B):** The patients’ information showed no significant difference in gender, age, and stage. The tumor size in Rb(+)Myc(+) seemed smaller than the other two groups, but showed no significant difference. **(C):** The survival rate in Rb(−)Myc(+) group was significant lower than the Rb(−)Myc(−) and Rb(+)Myc(+) groups. **(D):** The survival rate was significantly different in stage II, but not in stages I and III. Bars = 100 μm. Abbreviation: Rb, retinoblastoma.

In 72 tissue spots from primary OS patients, 47 cases (62.3%) were Rb(−)Myc(+), 15 cases (20.8%) were Rb(+)Myc(+), and 10 cases (13.9%) were Rb(−)Myc(−), whereas there were no cases were Rb(+)Myc(−), consistent with our findings that Rb silencing and c‐Myc overexpression were important for transformation of MSCs into OS‐like cells (Fig. 6A). Although, there was no significance difference in age, gender, or stages among different immune‐histological phenotypes (Fig. 6B), the survival rate was significantly different among different phenotypes, with the highest in the Rb(+)Myc(+) group, moderate in Rb(−)Myc(−) group, and the lowest in the Rb(−)Myc(+) group (Fig. 6C). When the survival rate was analyzed according to stages, we further found that the significance of difference was only observed in stage II patients (Fig. 6D). Moreover, the primary tumor size was lower in Rb(+)Myc(+) group compared with other types, albeit no significance was found. These data together suggest that Rb silencing and c‐Myc overexpression were important in clinical OS development and survival determination.

## Discussion

Although there is increasing evidence that MSCs have an important role in malignant transformation and cancer initiation, the link is still controversial. Some studies have successfully induced neoplastic transformation in human MSCs 41, 42, but no models have so far unequivocally demonstrated the transformation of human MSCs to OS. For example, the spontaneous transformation of human MSCs after long‐term culture was later proven to be due to cross‐contamination by other malignant cells 43, 44. From the review of Mutsaers and Walkley 45, genetic modification of preosteoblasts, rather than human MSCs (hMSCs), more readily induced OS, suggesting that preosteoblasts, rather than hMSCs, represent the ontological origins. OS is known as an osteoblastic‐type tumor that showed osteogenesis features. In the present study, genetic modification successfully transformed primary MSCs to OS‐like cells, suggesting MSCs as one of the potential origins of OS. This study demonstrates that OS‐like cells that are transformed from human MSCs have the ability to form xenotopic, orthotopic, and metastatic tumors in mice models and show a similar gene expression profile to that of human primary OS cells. The transformed SiRb‐OeMyc cells also change in the CD surface markers, increase the expression of pluripotent genes, increase the ability to form spheres in a serum‐free medium, and increase the ability to initiate primary and secondary tumor formation. These data suggest that human MSCs can be successfully transformed into tumor initiation cells by using a combination of Rb knockdown and c‐Myc overexpression. Notably, in murine models, various studies have shown that genetic modifications on stem cells or osteoblastic cells 11, 12, 14 induced tumorigenic transformation. However, the similar genetic modification to the murine model, such as combination silence of p53 and Rb in human MSCs, could not successfully induce transformation. The novelties of the present study include showing the transformation of human MSCs to osteosarcoma‐like cells by combination of Rb inhibition and c‐Myc overexpression.

The most common genes that are mutated in OS patients include tumor suppressor genes, such as p53 or Rb, and oncogenes, such as c‐Myc or Ras 15, 16, 46, 47. These genes have been studied for the transformation of MSCs to OS. In mice studies, conditional knockout of both p53 and Rb in the osteoblast lineage of mouse models in vivo has been shown to spontaneously induce OS tumors 14, 48. However, knockout of p53 or of both p53 and Rb in mouse adipose‐derived or bone marrow‐derived MSCs initiates the formation of leiomyosarcoma in vitro 49. The type of sarcoma initiated by mouse bone marrow‐derived MSCs, upon knockout of these genes, also depends on the differentiation lineage and the stage 49. In contrast, single or combined knockdown of these genes produces no transformation in human MSCs. It is also shown that individually, c‐Myc or Ras overexpression produces “oncogenic senescence” in human MSCs, as previously reported 50. Although many studies have successfully transformed mouse MSCs into OS‐like cells, this study is the first to transform human MSCs into OS‐like cells with the ability for tumorigenesis by combination of two complementary events. However, the underlying mechanism still shows a discrepancy between human and rodent studies, and the reason for this still remains unclear. Previous results for the expansion of human and mouse MSCs in vitro may explain this. Human MSCs have more stable chromosomes after long‐term expansion in a low‐density culture 27 compared with mouse MSCs, which undergo changes in the number of chromosomes, even after one to two passages through a low‐density culture 25, so there is a greater possibility of malignant transformation. Notably, the protein levels of p53 were lower in parental cells compared with transformed OS‐like cells, indicating that the absence or disruption of p53 does not play a role in c‐Myc overexpression and Rb knockdown‐induced tumorigenic transformation of human MSCs in the present study. In this study, we investigated whether overexpression of c‐Myc/Ras and/or disruption of p53 and Rb induced tumorigenic transformation of normal MSCs. We defined that only overexpression of c‐Myc and disruption of Rb consistently induced tumorigenic transformations of human MSCs from four individuals into OS‐like cells.

Notably, our data shown in human MSCs are consistent with and supported by a previous study, which shows that mouse MSCs derived from mice with a homozygous deletion of the *Ink4a/Arf* locus (Ink4aKO mice) can be transformed into mouse OS through overexpression of c‐Myc 47. Similarly, mouse OS cell clones derived from this model also decreased in the ability to differentiate into adipogenic cells. The Rb pathway is generally recognized as one of the most important pathways in cell proliferation and differentiation, being associated with many human cancers 51, 52. However, whether there are different roles for the Rb or c‐Myc pathways between these two human and mouse OS models remains unclear. Further studies are thus required to determine whether these OS models require the similar signaling pathways in transforming MSCs into OS‐like cells.

In order to characterize the tumors formed by SiRb‐OeMyc, the expression profiles of several sarcoma‐specific markers were studied. In the intraosseous tumors formed by SiRb‐OeMyc cells, osteoid tissue, cells that tested positive for ALP, osteonectin, and osteocalcin, and cells that tested slightly positive for desmin and α‐SMA were detected, but no cells that tested positive for PPARγ, h‐caldesmon, FLI‐1, and S100, which indicates that tumors that are formed by SiRb‐OeMyc are OS, but are not likely to be leiomyosarcoma, liposarcoma, Ewing tumor, or chondrosarcoma. Serakinci et al. demonstrate that telomerized hMSC (hMSC‐TERT20) underwent transformation 41. Although they did not claim the tumor type of the transformed hMSC‐TERT20 was OS, these tumors contained osteoid and expressed α‐SMA and CD99 41, indicating the similarity to OS. Similarly, tumors formed by SiRb‐OeMyc cells also formed osteoid tissues and expressed α‐SMA and CD99. Moreover, tumors formed by SiRb‐OeMyc cells were positive for ALP, osteonectin, and osteocalcin, heterogeneously positive for desmin and α‐SMA, the sarcoma marker, and negative for FLI‐1, S100, PPARγ, and h‐caldesmon, excluding the possibility of Ewing sarcoma, chondrosarcoma, liposarcoma, and leiomyosarcoma. These data together indicated that tumors formed by SiRb‐OeMyc cells were OS‐like and are similar to that formed by transformed hMSC‐TERT20. Subcutaneous tumors formed by SiRb‐OeMyc cells also have a pattern that is similar to that of intraosseous tumors, which excludes the possibility that the development of an OS tumor after intraosseous injection of SiRb‐OeMyc is affected by the intraosseous environment. These data suggest that SiRb‐OeMyc cells contain OS‐like cells and that they have the ability to form OS tumors in xenotopic and orthotopic areas.

Consistent with the ability to form OS in vivo, SiRb‐OeMyc cells in vitro readily showed osteoblastic differentiation, yet had decreased ability to differentiate into adipocytes. The effect of Rb knockdown on the determination of the fate of human MSCs is supported by the results of a previous study, which show that Rb is required for adult adipocyte differentiation 53. In contrast to the results of these studies, Rb deletion in mouse MSC lineage increases the adipogenic potential and decreases the osteogenic potential, which results in the formation of a liposarcoma 12. These findings and those of previous studies show a conflicting role for Rb in the determination of the fate of MSCs. This study does not determine the detailed mechanism that Rb mediates to determine lineage differentiation or the mechanism that causes the discrepancy between human and mouse MSCs. However, it is speculated that the discrepancy in the role of Rb knockdown or knockout on the determination of the fate of human and mouse MSCs may be because of several reasons. For example, the overexpression of c‐Myc, combined with Rb knockdown, in the model proposed by this study has been reported to enhance the expression of osteogenic genes in human MSCs 54.

Microarray analysis of the transcriptomes of SiRb‐OeMyc cells, their corresponding parental MSCs, primary OS cells, and MSCs from OS patients showed that the gene expression profiles of SiRb‐OeMyc and primary OS are located far away from parental and patients’ derived MSCs, and are scattered at different locations, reflecting the diversity of tumor cells. The GO analysis of the changes in gene expression shows that the genes that are associated with cell differentiation, development, or growth are upregulated or downregulated, after the transformation of MSCs by a combination of Rb knockdown and c‐Myc overexpression, which indicates that the malignant transformation of MSCs is accompanied by a change in stem cell properties, including the proliferation capacity and the differentiation potential. The molecular networks that dominate in SiRb‐OeMyc cells give an insight into the relationship between c‐Myc and p53 and other signaling molecules, such as VEGF, β‐catenin, and TGF‐β1.

We have recently shown that MSCs at early passage increased in the total and phosphorylated levels of Rb compared with MSCs at late passage, which upregulates DNA methyltransferase 1 to induce DNA methylation of cell cycle regulator genes, thereby stimulating proliferation and maintaining stem cell properties 55. Knockdown of Rb induces premature senescence in MSCs (supplemental online Fig. 7A). Previously, overexpression of an oncogene, such as c‐Myc, has been shown to induce DNA hyperreplication, which triggers a DNA damage response and subsequently causes cell senescence 56, 57( supplemental online Fig. 7B). The present study unexpectedly demonstrates that a combination of Rb silencing and c‐Myc overexpression induces MSC transformation rather than senescence, which depends on the GSK‐3β/β‐catenin pathway (supplemental online Fig. 7C).

Although we conducted transfection of the cells with constructs carrying H‐ras, we do not have supportive evidence to show that the ras constructs used here were functional. Because the cells transfected with ras constructs could not survive or proliferate enough to get enough cell lysates for Western blotting analysis, we did not confirm the biological activities of these constructs. To answer these questions, future positive controls, such as focus formation assay in NIH‐3T3 cells with the same genetic modification, should be performed in the future.

## Conclusion

Consistent with their roles in transformation of normal MSCs into OS‐like cells, our clinical data demonstrate that Rb silencing and c‐Myc overexpression are important in clinical OS development and survival determination. Thus, Rb silencing and c‐Myc overexpression may be considered targets for prevention and treatment of clinical OS patients. To accomplish this goal, further studies need to explore the underlying pathways that Rb and c‐Myc mediate to transform MSCs into OS‐like cells. Nevertheless, this is the first study to succeed in using genetic modification to achieve prompt transformation of human MSCs into osteosarcoma‐like metastatic cells, which provides a cell platform for future studies of the mechanisms for OS transformation, progression and treatment.

## Author Contributions

J.‐Y.W.: conception and design, collection and/or assembly of data, data analysis and interpretation, manuscript writing; P.‐K.W.: clinical consulting; P.C.‐H.C.: patient sample tissue array immunostaining and analysis; C.‐W.L.: data collection, assistance with experiments; W.‐M.C. and S.‐C.H.: experiment design, final approval of manuscript, correspondence.

## Disclosure of Potential Conflicts of Interest

S.‐C.H. has uncompensated employment and intellectual property rights, acts in a consultant/advisory role, receives honoraria, research funding, and stock options, and provides expert testimony. The other authors indicated no potential conflicts of interest.

## Supporting information

Supporting Information
