# Supplementary material for: Generation of Osteosarcomas from a Combination of Rb Silencing and c‐Myc Overexpression in Human Mesenchymal Stem Cells
Source: Stem Cells Transl Med. 2016 Sep 7;6(2):512–26. doi: 10.5966/sctm.2015-0226 (PMC5442803; doi:10.5966/sctm.2015-0226)
Supplement: Supplementary file 1 — Supporting Information [file SCT3-6-512-s001.pdf]

Supplemental Figures – Hung and Chen et al.

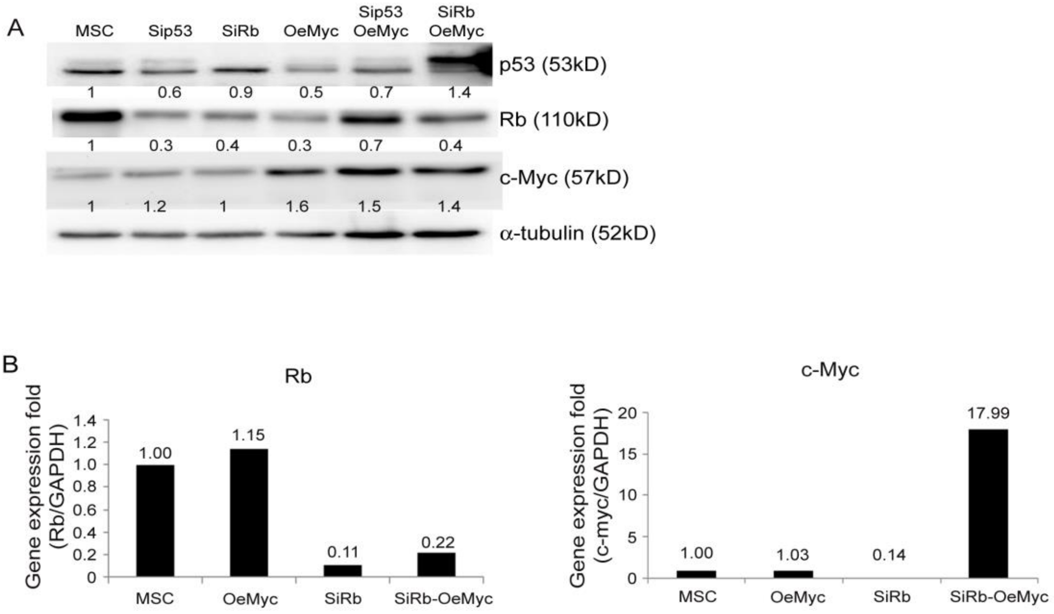

Figure S1. Confirmation of the genetic modification of either Rb or p53 and overexpression of c-Myc in human MSCs. (A) Western blot; (B) Quantitative RT-PCR.

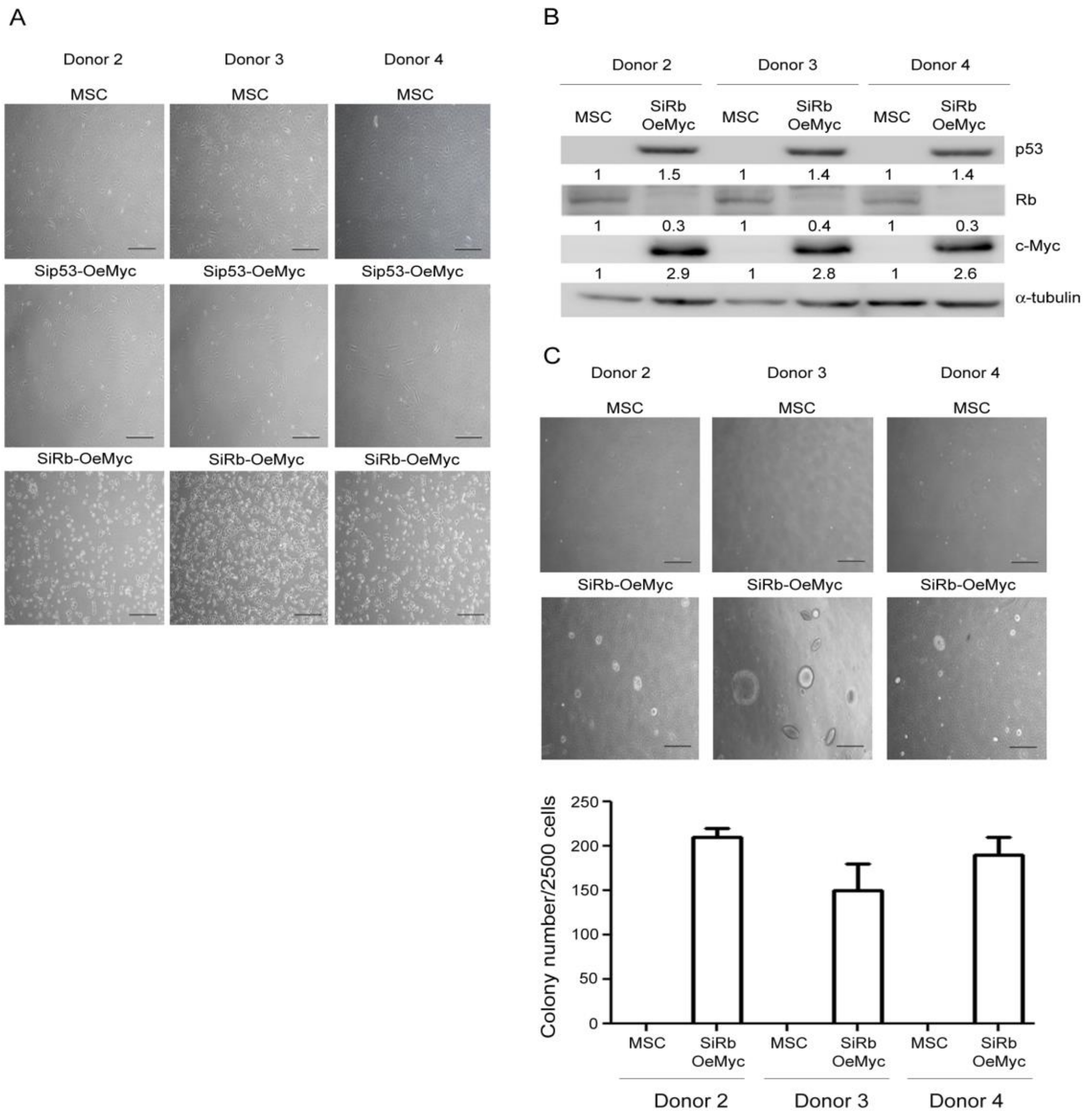

Figure S2. Silencing of Rb and overexpression of c-Myc in MSCs from another three individual donors also transformed human MSCs to tumor cells. (A) The morphology of parental, Sip53-OeMyc and SiRb-OeMyc cells from another three donors. Notably, Sip53-OeMyc ceased to grow. (B) The efficiency of silencing Rb and overexpression of c-Myc was confirmed by Western blotting. (C) The abilities of parental MSCs and SiRb-OeMyc cells to form colonies in soft agar assay. Upper panels show the representative pictures. Lower panels show the quantitative data (mean±SD). Bar = 100 μm.

A

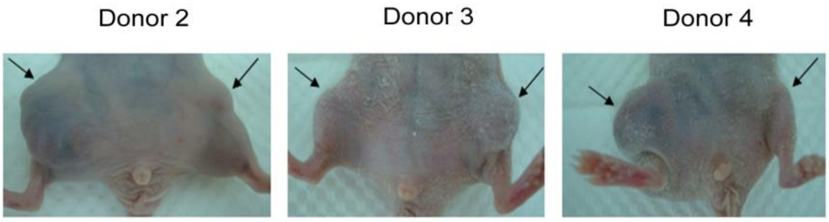

B

	Donor 2	Donor 3	Donor 4
W1	0/4	0/6	0/6
W2	0/4	0/6	0/6
W3	1/4	3/6	2/6
W4	1/4	3/6	2/6
W5	2/4	4/6	6/6
W6	4/4	6/6	6/6

C

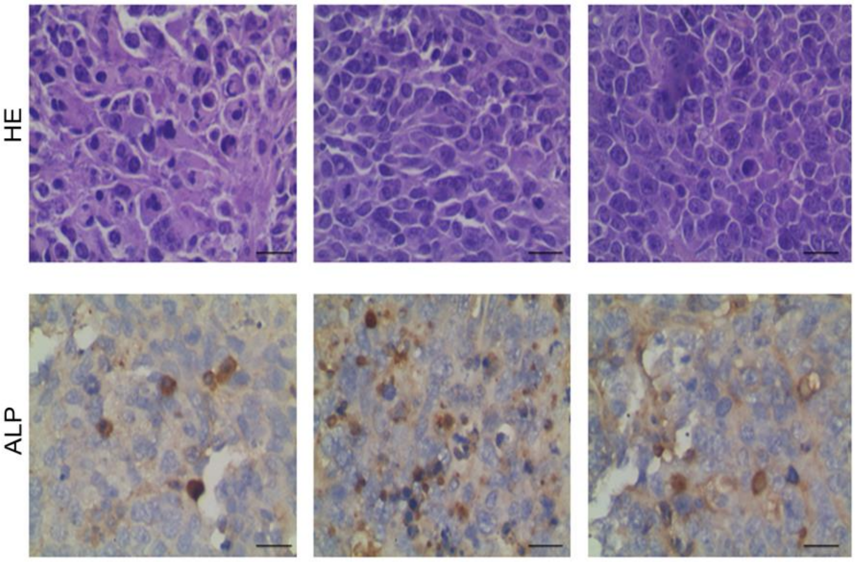

D

Tumor markers		siRb-OeMyc
Osteosarcoma	ALP	+
	osteonectin	+
	osteocalcin	+
Rhabdomyosarcoma	desmin	+
Leiomyosarcoma	α-SMA	+/-
	h-caldesmon	-
Ewing's sarcoma	FLI-1	-
Chondrosarcoma	S100	-
Liposarcoma	PPAR-γ	-

Figure S3. The SiRb-OeMyc cells from other three donors formed intraosseous OS-like tumors in nude mice. (A) Genetically altered MSC cells (10^7) were injected into tibia and formed tumor (arrows). (B) Tumor formation rates (n/6, n indicates numbers of tumor formed by six intra-tibial injection) were calculated up to 6 weeks. No tumor was formed by intra-tibial injection of parental MSCs for 16 weeks. (C) The HE staining and ALP immunohistochemistry demonstrated the OS feature. (D) The lists of antibodies used for immunohistochemistry. Bar = 100 μm.

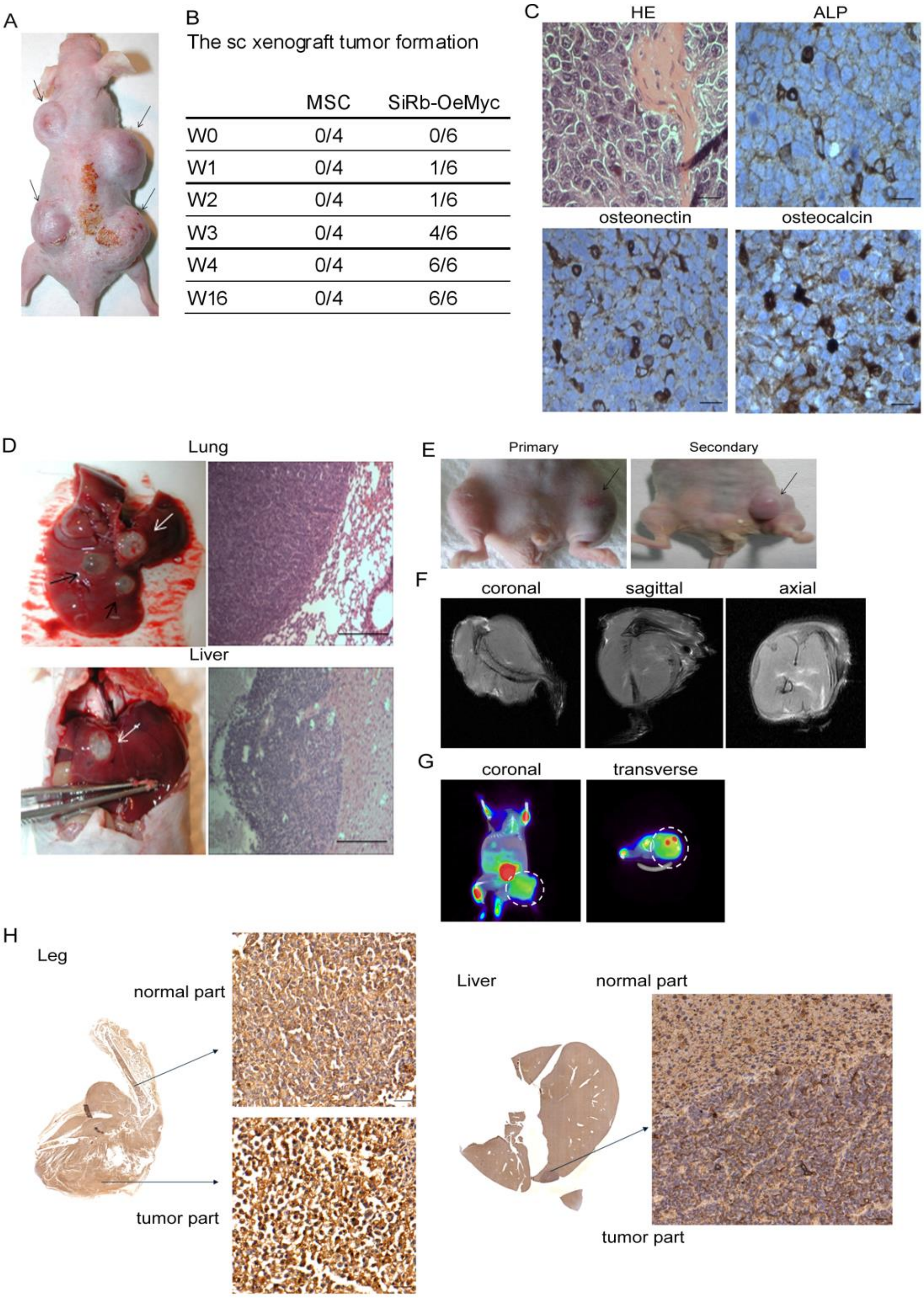

	MSC	SiRb-OeMyc
W0	0/4	0/6
W1	0/4	1/6
W2	0/4	1/6
W3	0/4	4/6
W4	0/4	6/6
W16	0/4	6/6

Figure S4. SiRb-OeMyc cells also form tumors in subcutaneous xenograft model and show metastasis and secondary-tumorigenesis ability. (A)SiRb-OeMyc cells formed tumors (arrows) by subcutaneous injection. (B) Theratiooftumorformation for each injection at the indicated time period after injection is shown. (W:week) (C) Hematocylin and eosin stain (HE) and immunohistochemistry show the subcutaneous tumors formed by SiRb-OeMyc have osteoid feature (HE staining) and expressed OS markers (ALP, osteonectin and osteocalcin). (D left two panels) The subcutaneous-injected cells metastasized to lung and liver and formed gross tumor nodules (arrows). (D right panels) HE staining shows tumor formation. (E) Cells isolated from SiRb-OeMyc-formed intraosseous tumor (arrow) maintained the ability to formed secondary intraosseous tumor (arrow). (F) The representative magnetic resonance imaging (MRI) and (G) micro-PET/CT show secondary intraosseous tumor (dotted line). Bar = 500 μm. (H) The tumors formed by SiRb-OeMyc cells in tibia were significantly positive for human nuclei (dark brown spots), while neighboring non-tumor parts were negative for human nuclei (blue spots) (left panel) and the liver nodule showed human nuclei negative (right panel). Bar = 100 μm.

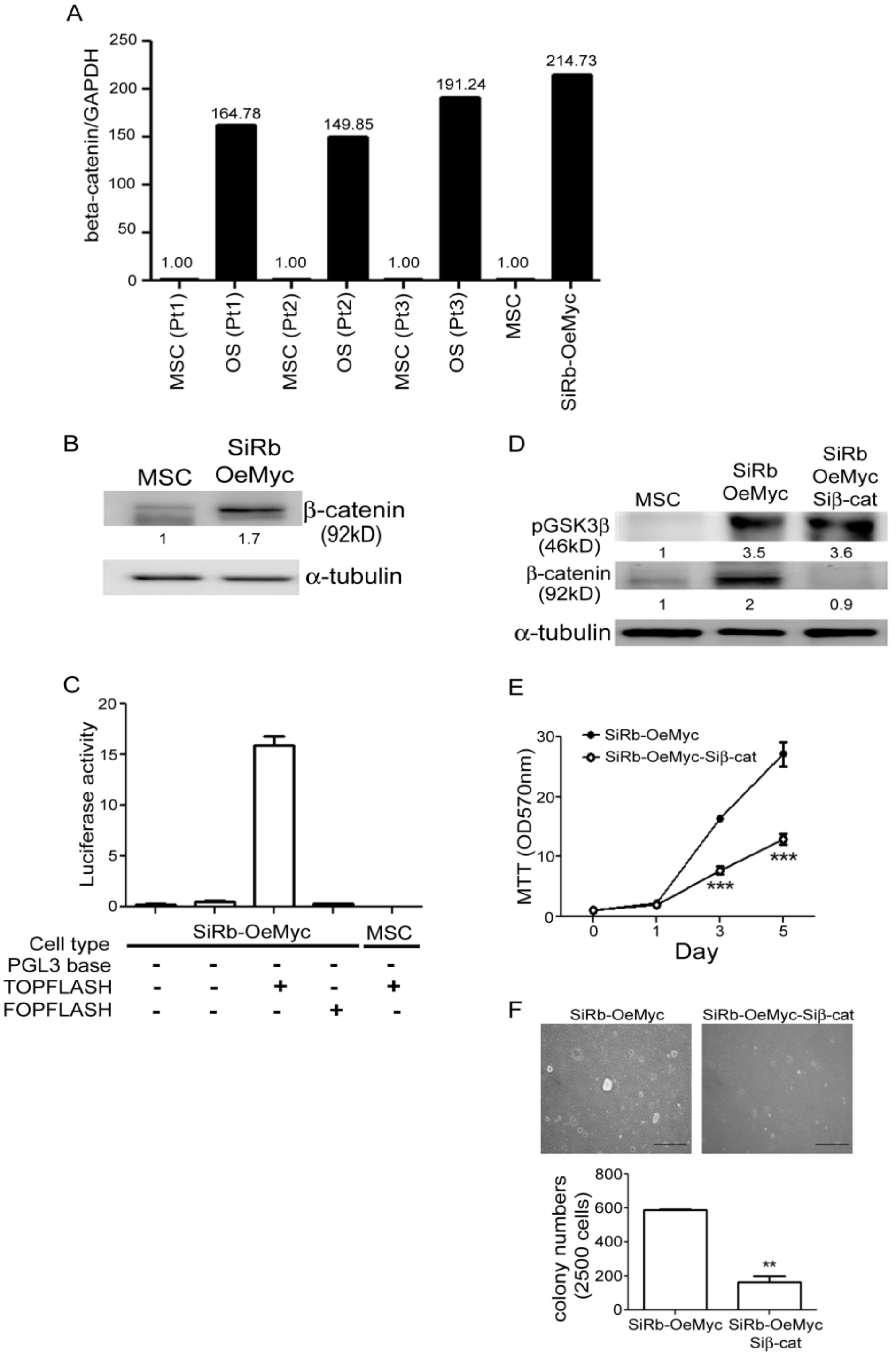
A
beta-catenin/GAPDH
1.00
164.78
1.00
149.85
1.00
191.24
1.00
214.73
MSC (Pt1)
OS (Pt1)
MSC (Pt2)
OS (Pt2)
MSC (Pt3)
OS (Pt3)
MSC
SiRb-OeMyc
B
MSC
SiRb OeMyc
β-catenin (92kD)
1
1.7
α-tubulin
C
Luciferase activity
Cell type
SiRb-OeMyc
MSC
PGL3 base
TOPFLASH
FOPFLASH
D
MSC
SiRb OeMyc
SiRb OeMyc Siβ-cat
pGSK3β (46kD)
1
3.5
3.6
β-catenin (92kD)
1
2
0.9
α-tubulin
E
SiRb-OeMyc
SiRb-OeMyc-Siβ-cat
MTT (OD570nm)
Day
***
***
F
SiRb-OeMyc
SiRb-OeMyc-Siβ-cat
colony numbers (2500 cells)
SiRb-OeMyc
SiRb-OeMyc Siβ-cat
**

Figure S5. β-catenin level and activity were upregulated in SiRb-OeMyc cells compared to parental MSCs (MSC). (A) The mRNA expression level of β-catenin was assayed by quantitative realtime PCR with SYBR system. SiRb-OeMyc cells and primary OS cells had higher β-catenin mRNA levels than their parental MSCs or primary normal MSCs. (B) The protein expression level of β-catenin was analyzed by Western blotting and the result showed higher protein level of β-catenin in SiRb-OeMyc cells. (C) The TOP/FOP-FLASH luciferase reporter assay was performed by examining the reporter activity of β-catenin. TOP-FLASH is wild-type Tcf-binding site that is activated by the binding of β-catenin, while FOP-FLASH is a reporter plasmid containing mutant Tcf-binding site and serves as a negative control. The result showed higher β-catenin activity in SiRb-OeMyc cells than in their parental cells. (D-F) SiRb-OeMyc cells without (transfected with scrambled shRNA) or with β-catenin knockdown (Siβ-cat) were subjected to western blotting analysis for β-catenin signaling molecules and GSK-3β (D). Cell growth rate was performed by MTT assay in 96-well plates. The growth rate of SiRb-OeMyc-Siβ-cat cells were significantly slower than that of SiRb-OeMyc cells without β-catenin knockdown. (E). The soft agar colony formation assay was performed by seeding 2500 cells on soft agar, followed by the calculation of colony number 14 days later. Aggregates with cell numbers greater than 250 cells were recognized as colonies. These data showed silencing of β-catenin induced a significant decrease in soft agar formation (F). $^{**}P<0.01$ and $^{***}p<0.005$ compared to control SiRb-OeMyc cells. Bar =

500 μm.

A
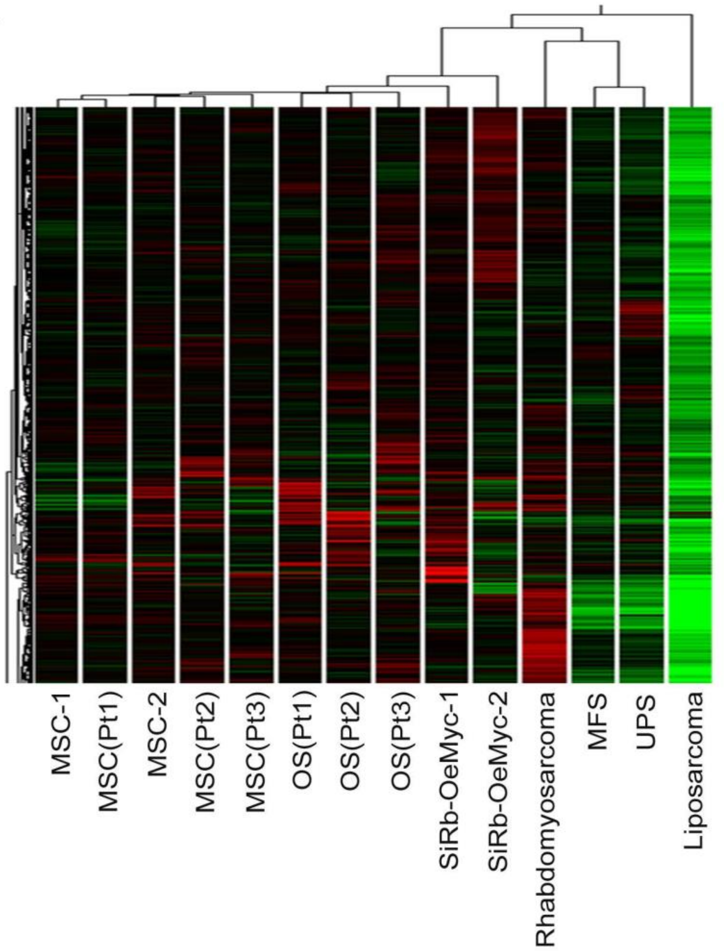
MSC-1
MSC(Pt1)
MSC-2
MSC(Pt2)
MSC(Pt3)
OS(Pt1)
OS(Pt2)
OS(Pt3)
SiRb-OeMyc-1
SiRb-OeMyc-2
Rhabdomyosarcoma
MFS
UPS
Liposarcoma

B
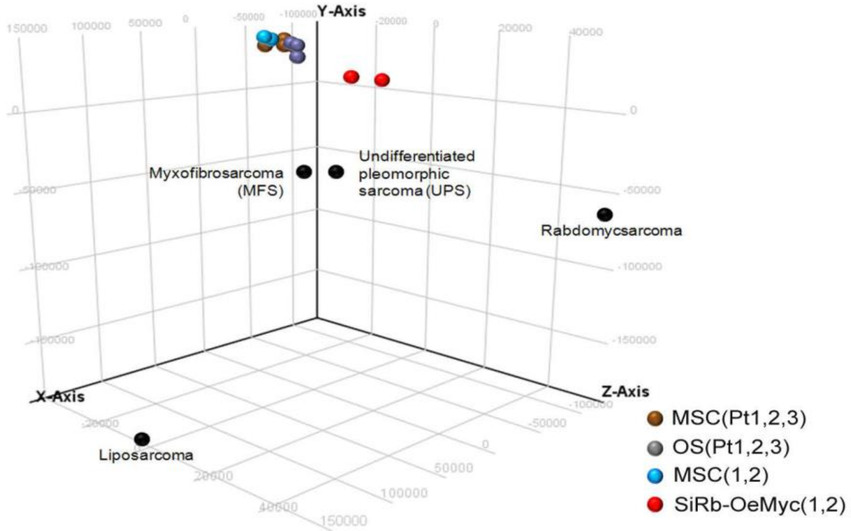
Y-Axis
X-Axis
Z-Axis
Myxofibrosarcoma (MFS)
Undifferentiated pleomorphic sarcoma (UPS)
Rabdomycsarcoma
Liposarcoma
MSC(Pt1,2,3)
OS(Pt1,2,3)
MSC(1,2)
SiRb-OeMyc(1,2)

Figure S6. The SiRb-OeMyc cells showed similar gene profile as human OS rather than other bone tumor. (A) Analyzing microarray data using cluster analysis. Heat map of genes with significant induction (red) or repression (green) showed similar pattern between SiRb-OeMyc (SiRb-OeMyc-1 and -2) and three primary OS cells isolated from OS patients (OS Pt-1, 2, 3) while parental MSCs (MSC-1 and -2) were close to primary MSCs from OS patients (MSC Pt-1, 2, 3) and were more difference to rhabdomyosarcoma, MFS, UPS, and liposarcoma. (B) The principle component analysis (PCA) shows close relationship between SiRb-OeMyc and primary OS cells rather than other bone tumors.

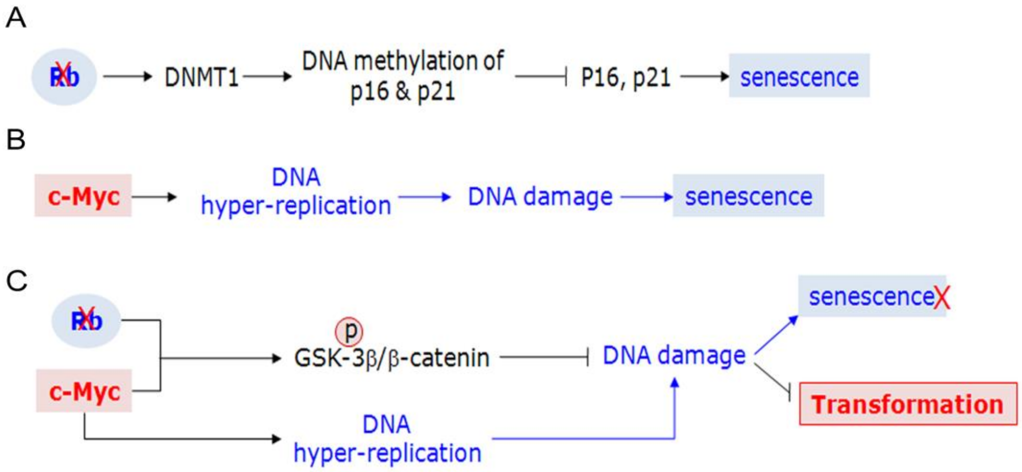

Figure S7. The mechanism pathway of the combination of Rb knockdown and c-Myc overexpression induced oncogenic transformation (A) Knockdown of Rb induces premature senescence in MSCs. (B) Overexpression of c-Myc induces DNA hyper-replication which triggers a DNA damage response and subsequently causes cell senescence. (C) The current study unexpectedly demonstrates that combination of Rb silencing and c-Myc overexpression induces MSC transformation rather than senescence, which depends on the GSK-3β/β-catenin pathway.
